# Supplementary material for: Identification of photoperiod-induced specific miRNAs in the adrenal glands of Sunite sheep (Ovis aries)
Source: Front Vet Sci. 2022 Jul 22;9:888207. doi: 10.3389/fvets.2022.888207 (PMC9354845; doi:10.3389/fvets.2022.888207)
Supplement: Supplementary file 1 [file Data_Sheet_1.ZIP › Supplementary file/miRDeep2 file.zip!/miRDeep2 file/Know_Structure/SP42A2/oar-mir-544.pdf]

miRBase precursor : oar-mir-544
Total read count : 512
oar-miR-544-5p read count : 486
oar-miR-544-3p read count : 19
remaining reads : 7

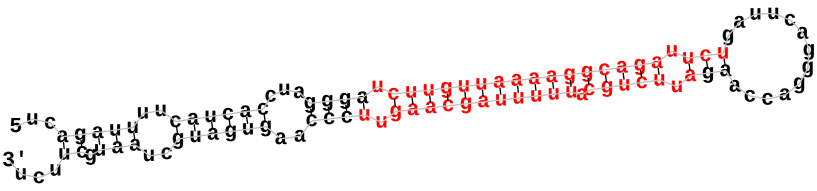

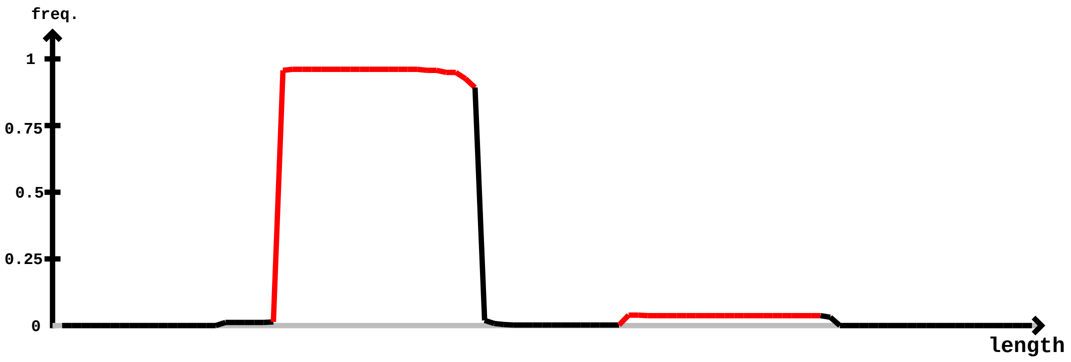


```
                                         oar-miR-544-5p
                                                                                oar-miR-544-3p
5'- ucagauuucaucaccuagggaucuuguuaaaaggcagauucugauucagggaccaagauucugcauuuuuagcaaguucccaagugaugcuaaugcuucu   -3'   exp
    ..(((((..((((((...((((.((((((((((((((((.(((..............))).))))).))))))))))).))))..))))))..))).))...   reads  mm  sample
    ...............uagggaucuuguuaaaaggca................................................................   2      0   seq
    ...............uagggaucuuguuaaaaggcaga..............................................................   4      0   seq
    ....................aucuuguuaaaaggcagauu............................................................   1      0   seq
    .....................ucuuguuaaaaggcagGuu............................................................   1      1   seq
    .....................ucuuguuaaaaggcagauu............................................................   10     0   seq
    .....................ucuuguuaaaaggcagauuc...........................................................   16     0   seq
    .....................ucuuguuaaaaggcagauuA...........................................................   1      1   seq
    .....................ucuuguuaGaaggcagauucu..........................................................   3      1   seq
    .....................ucuuguuaaaaggcagauucA..........................................................   29     1   seq
    .....................ucuuguuaaaaggcagUuucu..........................................................   1      1   seq
    .....................ucuuguuaaaaggUagauucu..........................................................   2      1   seq
    .....................uUuuguuaaaaggcagauucu..........................................................   1      1   seq
    .....................ucuuguuaaaaggcagauucu..........................................................   405    0   seq
    .....................ucuuguCaaaaggcagauucu..........................................................   1      1   seq
    .....................ucuuCuuaaaaggcagauucu..........................................................   1      1   seq
    .....................ucuuguuaaaaUgcagauucu..........................................................   1      1   seq
    .....................ucuuguuaaaaggcagaCucu..........................................................   3      1   seq
    .....................ucuuguuaaaaggcagauucuU.........................................................   4      1   seq
    .....................ucuuguuaaaaggcagauucuA.........................................................   2      1   seq
    .....................ucuuguuaaaaggcagauucugU........................................................   1      1   seq
    .....................ucuuguuaaaaggcagauucuAa........................................................   1      1   seq
    ......................cuuguuaaaaggcagauucu..........................................................   1      0   seq
    ......................cuuguuaaaaggcagauucugau.......................................................   1      0   seq
    ..........................................gauucaUggaccaagau.........................................   1      1   seq
    ........................................................auucugcauuuuuagcaaguu.......................   3      0   seq
    ........................................................auucugcauuuuuagcaaguuU......................   7      1   seq
    ........................................................auucugcauuuuuagcaaguuc......................   9      0   seq
```
